# Difficulty and Importance of Correct Diagnosis, and Especially in Certain Nervous Affections

**Published:** 1856-04

**Authors:** J. Washington Jones

**Affiliations:** Auburn, Alabama


### ARTICLE III.

*Difficulty and Importance of Correct Diagnosis, and Especially in Certain Nervous Affections.* By J. WASHINGTON JONES, M. D,, of Auburn, Alabama.

On the 21st of November last, at 8 o'clock, P. M., Edy, a negro girl, belonging to A. F. of this village, aged nineteen years, of robust constitution, and good general health, had menstruation regularly and freely, since the first appearance of that discharge, which took place some fifteen or eighteen months since, while engaged in the ordinary duties of house-servant, suddenly fell forward, striking her right cheek against a chair, and thus inflicting a considerable contusion. I saw her about half an hour after she was attacked, and at once decided that she was not (as the family supposed,) in an apoplectic condition—her pulse was natural, countenance calm and placid, except some, almost imperceptible, twitchings about the mouth, and a slight knitting of the brows; respiration hurried and laborious, but not stertorious; each expiration was attended with a whistling or snuffling sound, not unlike that made by a crying child; she was speechless, and apparently in a state of profound insensibility; eyelids closed, pupils neither dilated nor contracted, but stationary; hands and feet warm, and the head slightly above the natural temperature; universal tremulousness and rigidity of the muscular system, the latter so much so that when either of the lower extremities were supported, or raised from the bed by placing a hand beneath it, and in the popliteal region, it would remain for more than ten minutes perfectly unrelaxed and extended—the superior extremities were less rigid but more tremulous; no frothing at the mouth; no well-marked convulsions.

Was this a case of epilepsy or hysteria, or was it either? I succeeded in administering, though with great difficulty, the dysphagia being considerable, a portion of the tincture of lobelia, which happened to be at hand, but not enough to produce emesis, and directed sinapisms to the extremities, and an active cathartic enema.

Nov. 22d, 9 o'clock, A. M. Two hours after she took the lobelia, I learned that the system became relaxed, articulation and deglutition was perfectly restored, and she slept from that time until morning—the enema was not administered. This morning, some tremulousness, but no rigidity; pulse natural, and tenderness upon pressure over the hypogastric region; ordered an ounce of castor oil with a drachm of oil of turpentine.

23d, 10, A. M. The cathartic draught operated thoroughly, and the patient appears quite improved in all respects.

Such, and similar cases I have often seen, and in some instances, their diagnosis is well calculated to perplex the practitioner, and especially if he has had but a limited experience; indeed, I may say, that our oldest and wisest physicians are not unfrequently at a loss to prescribe judiciously, under such circumstances.

I attach no particular consequence to the treatment adopted in this case; nor do I believe that it was materially, if at all, instrumental in releiving the patient, and the chief object in communicating the case to the profession, is to aid in illustrating the importance of a more intimate knowledge of general and special physiology and pathology. The similarity of symptoms, by which we are unable to distinguish epilepsy and hysteria, have been very clearly summed up, and set forth by Foville—"There are two principal forms of each disorder. In each, one of these forms is *convulsive*, and the other is not. The non-convulsive form of epilepsy relates exclusively, to the *Sensorium;* it is characterized by vertigo, and by a suspension, (however brief and transitory,) of the mental forms. The non-convulsive form of hysteria, has but little apparent connection with the animal functions; its palpable phenomena consist in derangement of the organic functions of the thorax and abdomen. It is the ganglionic portion of the nervous system that seems chiefly disturbed."

In epilepsy, there is an entire loss of consciousness; the patient has no recollection of what has occurred during the paroxysm; the face is generally of a livid hue; a white, or red foam or froth usually collects about the mouth and lips; the convulsions are not uniformly the same on each side of the system; affecting the face and extremities more on one side than the other, and continue with but little intermission during the continuance of the fit, and an apparently strangling rattle attends respiration—the countenance is frightful; the eyelids partly open, and the eyeballs rolling in various directions; the pupils dilated; the mouth often drawn to one side, and grinding of the teeth; and not unfrequently, the tongue protruded between the teeth gashed and bleeding.

Whereas, during a fit of hysteria, though there is often an apparent loss of consciousness, it is not really so, and if so, it is only for a short time, and the patient (says Dr. Watson,) often is able to repeat (though she may not always choose to confess it,) what has been said by the bystanders, during the period when she seemed insensible. This is a point of distinction well worth remembering, for more reasons than one: it not only helps the diagnosis when the fact comes out, but it suggests certain cautions for ourselves; we must take care not to say anything by the bedside of an hysterical patient which we do not wish her to hear, and we may take advantage of her apparent unconsciousness, and pretend to believe in it, and speak of certain modes of treatment which she will not much approve of, but the very mention of which, may serve to bring her out of the fit.

In hysteria, there is generally considerable contortion of the body, with rapid and violent fluxion and extension of the extremities; the breathing deep, sighing, sobbing, and perhaps, an occasional outbreak of laughter; florid cheeks; eyelids shut and tremulous; pupils of about the natural size. Hysteria most frequently occurs in females; indeed, it is almost peculiar to the sex, and is usually connected, in some degree, with disturbance of menstruation.

Dr. Marshall Hall says, that the most reliable distinction between the two diseases is, "that in hysteria, much as the *larynx* may be affected, it is never closed, in epilepsy it is closed."

